# Genetic and Biochemical Analysis of Anaerobic Respiration in Bacteroides fragilis and Its Importance *In Vivo*

**DOI:** 10.1128/mBio.03238-19

**Published:** 2020-02-04

**Authors:** Takeshi Ito, Rene Gallegos, Leigh M. Matano, Nicole L. Butler, Noam Hantman, Matthew Kaili, Michael J. Coyne, Laurie E. Comstock, Michael H. Malamy, Blanca Barquera

**Affiliations:** a Center for Biotechnology and Interdisciplinary Sciences, Rensselaer Polytechnic Institute, Troy, New York, USA; b Department of Molecular Biology and Microbiology, Tufts University School of Medicine, Boston, Massachusetts, USA; c Division of Infectious Diseases, Brigham and Women’s Hospital, Harvard Medical School, Boston, Massachusetts, USA; d Department of Chemistry and Chemical Biology, Rensselaer Polytechnic Institute, Troy, New York, USA; e Department of Biological Sciences, Rensselaer Polytechnic Institute, Troy, New York, USA; University of Massachusetts Amherst

**Keywords:** *Bacteroides fragilis*, respiration, NQR, gut microbiota

## Abstract

In bacteria, the respiratory pathways that drive molecular transport and ATP synthesis include a variety of enzyme complexes that utilize different electron donors and acceptors. This property allows them to vary the efficiency of energy conservation and to generate different types of electrochemical gradients (H^+^ or Na^+^). We know little about the respiratory pathways in *Bacteroides* species, which are abundant in the human gut, and whether they have a simple or a branched pathway. Here, we combined genetics, enzyme activity measurements, and mammalian gut colonization assays to better understand the first committed step in respiration, the transfer of electrons from NADH to quinone. We found that a model gut *Bacteroides* species, Bacteroides fragilis, has all three types of putative NADH dehydrogenases that typically transfer electrons from the highly reducing molecule NADH to quinone. Analyses of NADH oxidation and quinone reduction in wild-type and deletion mutants showed that two of these enzymes, Na^+^-pumping NADH:quinone oxidoreductase (NQR) and NADH dehydrogenase II (NDH2), have NADH dehydrogenase activity, whereas H^+^-pumping NADH:ubiquinone oxidoreductase (NUO) does not. Under anaerobic conditions, NQR contributes more than 65% of the NADH:quinone oxidoreductase activity. When grown in rich medium, none of the single deletion mutants had a significant growth defect; however, the double Δ*nqr* Δ*ndh2* mutant, which lacked almost all NADH:quinone oxidoreductase activity, had a significantly increased doubling time. Despite unaltered *in vitro* growth, the single *nqr* deletion mutant was unable to competitively colonize the gnotobiotic mouse gut, confirming the importance of NQR to respiration in B. fragilis and the overall importance of respiration to this abundant gut symbiont.

## Abstract (precis)

*Bacteroides* species are abundant in the human intestine and provide numerous beneficial properties to their hosts. The ability of *Bacteroides* species to convert host and dietary glycans and polysaccharides to energy is paramount to their success in the human gut. We know a great deal about the molecules that these bacteria extract from the human gut but much less about how they convert those molecules into energy. Here, we show that B. fragilis has a complex respiratory pathway with two different enzymes that transfer electrons from NADH to quinone and a third enzyme complex that may use an electron donor other than NADH. Although fermentation has generally been believed to be the main mechanism of energy generation in *Bacteroides*, we found that a mutant lacking one of the NADH:quinone oxidoreductases was unable to compete with the wild type in the mammalian gut, revealing the importance of respiration to these abundant gut symbionts.

## INTRODUCTION

Studies of basic metabolic and energy-generating processes in bacteria have made important contributions to our understanding of how bacteria live in communities (1–3), adapt to changing environments (4), cause disease (3–6), and interact with their hosts (7, 8). Over the last decade, the microbiotas of the human gut have been intensely studied, revealing the importance of these microbial communities to human health and development (9–16). Despite all that we have learned, we still know relatively little about the central metabolic processes of many of the predominant bacterial members of this ecosystem.

*Bacteroides* is an abundant Gram-negative genus of the human intestinal microbiota, with its members predicted to stably colonize the host over a lifetime (17). *Bacteroides* species are saccharolytic bacteria that utilize complex dietary polysaccharides and host glycans present in the colon as their main carbon and energy sources. The ability of *Bacteroides* to harvest, degrade, and import these polysaccharides has been an area of intense study, yielding a wealth of important data (reviewed in reference 18). However, we know much less about how energy is generated from these molecules.

Aerobic respiration and anaerobic respiration are major energy-generating pathways of bacteria and are also the primary pathways for recycling the essential redox substrate NADH. NADH is generated by oxidative pathways, such as glycolysis and the Krebs cycle, and must be recycled to NAD^+^ to serve as the substrate for these pathways (19, 20). In the initial step of respiration, NADH dehydrogenases (NADH:quinone oxidoreductases) transfer electrons from NADH to quinone at the cell membrane, thus recycling NADH to NAD^+^. In aerobic respiration, these electrons are then transferred from the reduced quinone to O_2_ by means of various cytochrome oxidases (21). During anaerobic growth, electrons can be transferred to other terminal electron acceptors, such as fumarate, nitrate, or sulfate, by the action of membrane-bound reductase enzymes (22–25). These electron transfer steps produce significant amounts of energy, and NADH dehydrogenases and cytochrome oxidases are typically able to conserve this energy by pumping either H^+^ or Na^+^ from the cytoplasm to the periplasm, forming transmembrane electrochemical gradients (20, 21, 26–31). These gradients provide a driving force for cations to return from the periplasm to the cytoplasm and thus supply power for cellular processes, including the transport of substrates and the generation of ATP by membrane-bound ATP synthases (21, 32–35).

Three different NADH:quinone oxidoreductases have been described in bacteria, and each has different catalytic functions, cofactors, and evolutionary origins (reviewed in reference 20). H^+^-pumping NADH:ubiquinone oxidoreductase (NUO), or complex I, is the best studied due to its presence in mitochondria, where it is the only NADH:quinone oxidoreductase and is thus essential for energy generation (20). NUO is widely present in bacteria, where it is a 10- to 14-subunit protein complex with a flavin mononucleotide (FMN) and several iron-sulfur centers as cofactors for redox reactions (36, 37). As NUO transfers electrons from NADH to quinone, it conserves energy by pumping H^+^ from the cytoplasm to the periplasm (38). Another NADH dehydrogenase that is present in fewer bacterial species and that is somewhat sporadically distributed (39) is Na^+^-pumping NADH:quinone oxidoreductase (NQR). This protein complex is comprised of six subunits with several flavins and an iron-sulfur cluster as redox cofactors (40). In many bacteria, NQR has been shown to conserve energy during electron transfer by pumping Na^+^ across the membrane (41, 42). The third described NADH dehydrogenase involved in respiration, NADH dehydrogenase II (NDH2), is a single membrane-associated protein that binds flavin adenine dinucleotide (FAD) as a redox cofactor (43). NDH2 does not pump ions across the membrane during electron transport and therefore does not conserve energy, but it may function to recycle NADH to NAD^+^ under conditions of high membrane potential (43).

Few studies have analyzed respiration in *Bacteroides* species (44–49). It has been shown that under anaerobic conditions, fumarate can serve as a terminal electron acceptor, with fumarate reductase (FRD) transferring electrons from the reduced quinone, producing succinate (47, 48, 50). To our knowledge, no studies have analyzed NADH:quinone oxidoreductase activity in *Bacteroides* species, and the complexes involved in this important energy generation step in *Bacteroides* species are unknown. Phylogenetic studies have identified the genes for both NUO (51) and NQR (39) in the *Bacteroides* genome, but no detailed biochemical or mutational analyses have demonstrated the involvement of these complexes in respiration. NQR activity has been identified in the related organism Prevotella copri, where a study of central carbon metabolism found NADH:quinone oxidoreductase activity in isolated cell membranes (52). This activity was attributed to NQR, as several of the genes coding for NUO subunits are absent in Prevotella copri. Similar results have also been reported for Prevotella bryantii (53). In the oral bacterium Porphyromonas gingivalis, the RprY response regulator positively activates NQR by interacting with the promoter upstream of the *nqrA* gene (54), and under conditions of oxidative stress, the production of RprY decreases, as does the expression of *nqrA* (55). In addition, the RprY regulator of P. gingivalis was shown to bind the promoter region of the *nqr* operon of Bacteroides fragilis, suggesting that the regulation of this operon is similar in these two bacteria.

In addition to anaerobic respiration, *Bacteroides* species are also capable of aerobic respiration. When *Bacteroides* strains are grown under nanaerobic conditions (0.05 to 0.15% oxygen), similar to the conditions that may be present at the mucosal lining of the gut (56), oxygen can serve as the terminal electron acceptor. *Bacteroides* species contain cytochrome *bd* oxidase, a high-affinity oxidase that functions under low-oxygen conditions, transferring electrons from reduced quinone to oxygen, producing water (21, 57). During nanaerobic respiration in *Bacteroides*, cytochrome *bd* oxidase contributes to the proton motive force during electron transfer, thereby conserving energy (58).

Here, we show that B. fragilis has orthologs of all three diverse NADH:quinone oxidoreductases, NUO, NQR, and NDH2. Through the creation and analysis of single and double deletion mutants, combined with activity measurements of isolated membrane fractions, we show that both NQR and NDH2 have functional NADH dehydrogenase activity but that NUO does not. We found that a mutant lacking NQR was unable to compete with wild-type (WT) bacteria in a gnotobiotic mouse competitive colonization model. In contrast, a mutant lacking NUO showed no competitive colonization defect, while a mutant lacking NDH2 had a modest colonization defect. This is the first study demonstrating the importance of NQR to bacterial fitness in the colonization of a mammalian host. On the basis of these results, we propose a new paradigm of respiration in *Bacteroides* species that is more complex than previously appreciated, with NQR playing a critical role in energy generation.

## RESULTS

### Identification of putative NADH:quinone oxidoreductase genes in B. fragilis.

**FIG 1:**
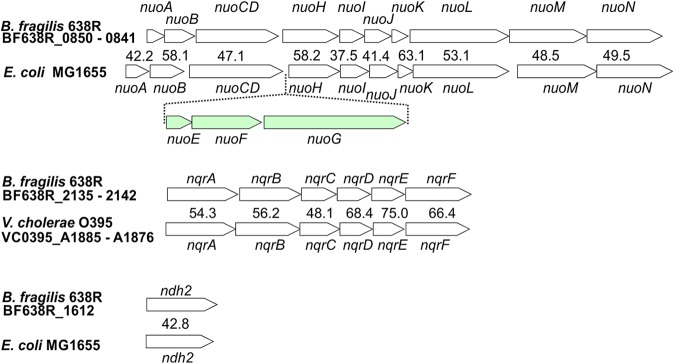
Identified operons of B. fragilis encoding predicted NADH:quinone oxidoreductases. The respective operons from E. coli or V. cholerae (*nqr*) are shown below each region, and the percent similarities of the products of the genes from these strains are listed. The *nuo* operon of B. fragilis is lacking three genes, shown in green, present in the *nuo* operon of E. coli.

Analysis of the genome sequence of B. fragilis strain 638R revealed that it contains each of three described types of NADH:quinone oxidoreductases: NUO (BF638R_0850-0841), NQR (BF638R_2136-2141), and NDH2 (BF638R_1612). Figure 1 shows the organization of each of these genes/operons compared to their organization in the corresponding regions of Escherichia coli for *nuo* and *ndh2* and of Vibrio cholerae for *nqr*, as E. coli lacks *nqr*. The three genes shown in green in the E. coli *nuo* operon but missing in the corresponding B. fragilis operon code for the soluble portion of the enzyme complex that includes the NADH binding site. The presence of genes for these three different types of electron transfer complexes suggests that B. fragilis has a respiratory chain more complicated than previously appreciated, containing enzymes that have been shown in other organisms to pump H^+^ (NUO) or Na^+^ (NQR), as well as NDH2, which was not previously reported in the *Bacteroidales*.

### Measurement of NADH:quinone oxidoreductase activity of isolated membranes.

**FIG 2:**
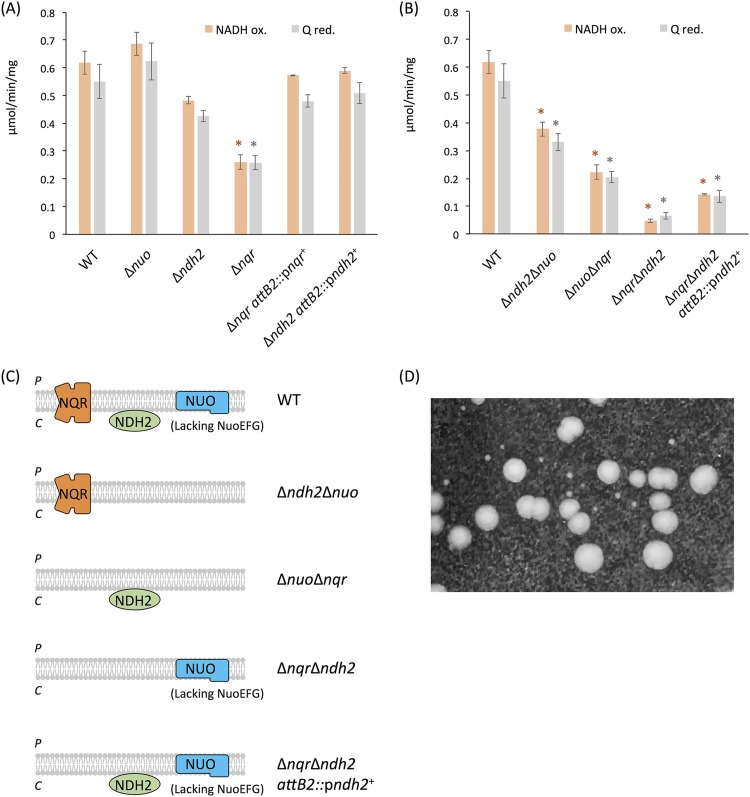
(A and B) NADH:quinone oxidoreductase activity in membranes of the B. fragilis WT (A and B), the single deletion mutant strains (the Δ*nuo*, Δ*ndh2*, and Δ*nqr* strains) and complemented strains (the Δ*nqr attB2*::p*nqr^+^* and Δ*ndh2 attB2*::p*ndh2^+^* strains) (A), and double deletion mutant strains (the Δ*ndh2* Δ*nuo*, Δ*nuo* Δ*nqr*, Δ*nqr* Δ*ndh2*, and Δ*nqr*Δ *ndh2 attB2*::p*ndh2^+^* strains) (B). The rates of NADH oxidation (NADH ox.; orange) and menadione (vitamin K_3_) reduction (Q red.; gray) using NADH were measured. Mean values (*n* = 3 to 5 experiments) are presented, and error bars show the standard errors. Data were analyzed by Dunnett’s test. *, *P* < 0.05 between the wild type and each mutant strain. (C) Diagram showing the enzyme that is present in each double mutant, i.e., the enzyme whose activity is measured. The letters P and C at the left-hand side of the membrane indicate the periplasm and cytoplasm, respectively. (D) Colony size comparison between the Δ*nqr* Δ*ndh2* mutant (small colonies) and the Δ*nqr* single-deletion-mutant parent strain (large colonies). The photograph was taken after 5 days of growth.

To study the role of each of these enzymes in the respiration of B. fragilis, we made mutants with an internal deletion in each gene/operon, performed biochemical activity measurements, and compared the NADH:quinone oxidoreductase activity in each of the mutants to that in the WT strain. Figure 2 shows measurements of NADH:quinone oxidoreductase activity measured in two ways: by the steady-state rates of NADH oxidation and by the reduction of menadione. For the *nuo* deletion mutant, there was no statistically significant difference in the activities in the mutant compared to that in the WT. This was the result that we predicted, based on the lack of *nuoEFG* in the *nuo* operon. In contrast, in the *ndh2* and *nqr* deletion mutants, the activities were significantly decreased and were approximately 78% and 42% of the WT values, respectively. These results strongly suggest that under the growth conditions used in these experiments, the NADH:quinone oxidoreductase activity of B. fragilis is a function of NQR and NDH2 in a roughly 2:1 ratio, with no contribution from NUO, as predicted based on the lack of *nuoEFG*. Full NADH:quinone oxidoreductase activity was restored in the *nqr* and *ndh2* complemented strains (Fig. 2A), indicating that the activity lost was due to these deletions.

Three double deletion mutants were created to provide more specific information about the contribution of each individual enzyme complex. As illustrated in Fig. 2B, each double deletion mutant could synthesize only one of the three enzymes, allowing measurements to focus on the activity of the one remaining enzyme (Fig. 2C). The activity in the Δ*ndh2* Δ*nuo* strain was approximately 61% of that in the WT, which was similar to that of the *ndh2* single deletion mutant, as expected, since we showed that the *nuo* single deletion mutant had full NADH:quinone oxidoreductase activity (Fig. 2A). Activity in the Δ*nuo* Δ*nqr* strain was approximately 36%, which was similar to that in the *nqr* single deletion mutant. These results strengthen our conclusion that NUO does not contribute to NADH:quinone oxidoreductase activity, as predicted. Furthermore, the Δ*nqr* Δ*ndh2* deletion mutant exhibited essentially no measurable activity (Fig. 2B), supporting the suggestion that NUO is not an NADH:quinone oxidoreductase in B. fragilis.

The Δ*nqr* Δ*ndh2* mutant was created by making an *ndh2* deletion in the Δ*nqr* strain. During resolution of the *ndh2* cointegrate, we noticed that both normal and small-size colonies were produced and determined that the small colonies were those of the Δ*nqr* Δ*ndh2* mutant (Fig. 2D). This growth defect likely results from the greatly reduced ability of this mutant to recycle NADH. In addition, this mutant likely obtains most of its energy by substrate-level phosphorylation, which yields less ATP per molecule of glucose, which may also contribute to the slower growth. When the *ndh2* operon was added back to the Δ*nqr* Δ*ndh2* mutant, the NADH:quinone oxidoreductase activity was recovered (Fig. 2B).

### Use of NADH analogs.

**FIG 3:**
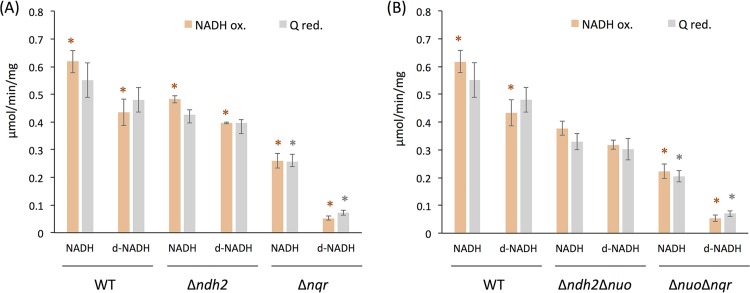
NADH:quinone oxidoreductase activities in membranes of the B. fragilis WT (A and B), the single deletion mutant strains (the Δ*ndh2* and Δ*nqr* strains) (A), and the double deletion mutant strains (the Δ*ndh2* Δ*nuo* and Δ*nuo* Δ*nqr* strains) (B), comparing NADH and deamino-NADH (d-NADH) as substrates. The rates of NADH oxidation (NADH ox.; orange) and menadione (vitamin K_3_) reduction (Q red.; gray) using NADH or d-NADH were measured. Mean values and standard errors are from 3 to 5 experiments. Data were analyzed by an unpaired *t* test (*, *P* < 0.05) between NADH and d-NADH for each strain.

To further analyze the NADH:quinone oxidoreductase of B. fragilis, we utilized deamino-NADH (d-NADH), a substrate analog of NADH (Fig. 3). d-NADH has been widely reported in other species to donate electrons to NUO and NQR but not to NDH2 (59–61). This specificity makes it possible to discriminate between NDH2 activity and the other NADH dehydrogenase activities by comparing the activities with the two different substrates. To confirm that d-NADH has the same specificity in B. fragilis, we first measured the activities with each of the substrates in the two double deletion mutants that retained only one type of NADH:quinone oxidoreductase activity, the Δ*ndh2* Δ*nuo* mutant, which contains only *nqr*, and the Δ*nuo* Δ*nqr* mutant, which contains only *ndh2*, as well as the WT. In the WT and the mutant containing only *nqr*, the activities with d-NADH were slightly lower than those with NADH, consistent with reports that d-NADH is a slightly less efficient substrate. However, in the mutant containing only *ndh2*, the activity was almost completely absent. Thus, d-NADH can be used in B. fragilis to discriminate the NDH2 activity in the presence of other NADH:quinone oxidoreductases.

Based on this finding, we then used NADH and d-NADH to analyze single deletion mutants. The activity of the Δ*ndh2* mutant was approximately the same with either NADH or d-NADH, whereas the Δ*nqr* mutant was active with NADH but had very low activity with d-NADH. These results are another confirmation that B. fragilis NUO does not use NADH as an electron donor.

### *In vitro* growth analysis of single and double deletion mutants.

**FIG 4:**
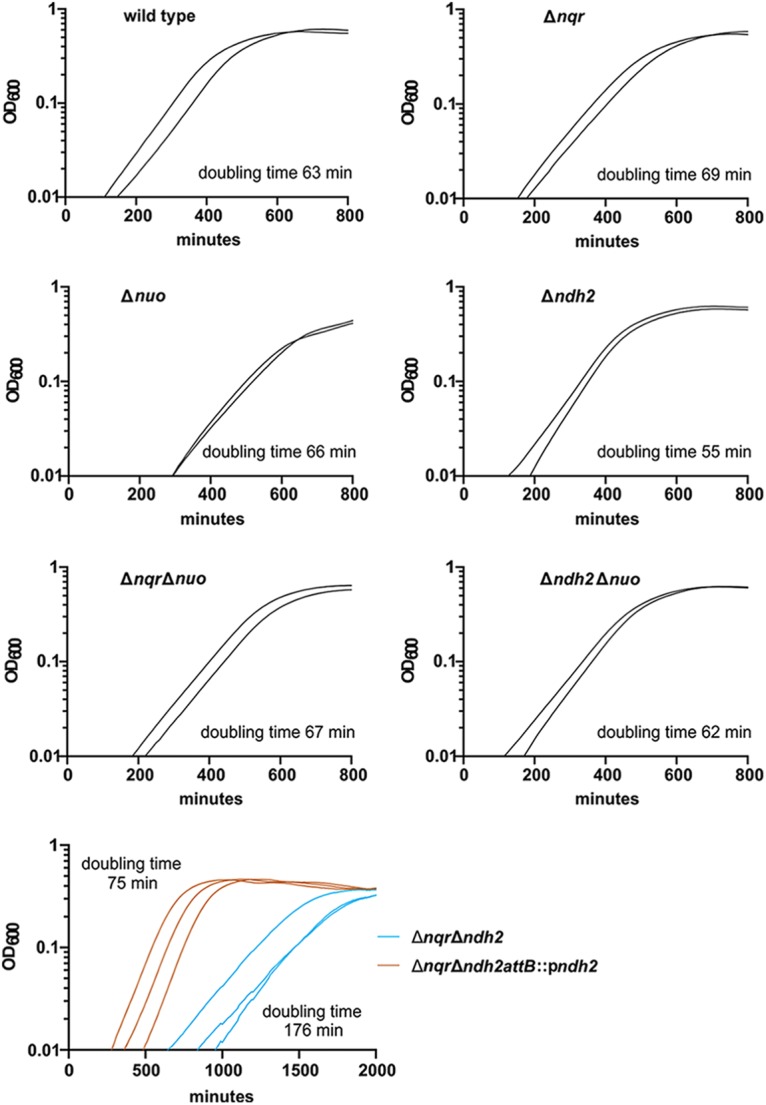
Growth curves for the WT, the single and double deletion mutants, and the Δ*nqr* Δ*ndh2 attB2*::p*ndh2^+^* strain in rich medium (BHIS medium) under anaerobic conditions. The growth of each strain (in duplicate or triplicate) was followed as the change in the absorbance at 600 nm in a BioTek plate reader. Doubling times were calculated as outlined in the Materials and Methods section, with all growth data being supplied in Table S1 in the supplemental material.

We investigated the growth characteristics of the single and double deletions in rich medium under anaerobic conditions (Fig. 4; see also Table S1 in the supplemental material). With the exception of the Δ*nqr* Δ*ndh2* mutant, all of the strains had doubling times within 13% of the doubling time of the WT and reached similar maximum values of the optical density at 600 nm (OD_600_) at the stationary-phase plateau. The Δ*nqr* Δ*ndh2* strain had a much longer doubling time (176 min) than the WT and an OD_600_ at the plateau of 70% of that of the WT. These changes were largely reversed when *ndh2* was reintroduced into the strain, yielding a much faster doubling time of 75 min (Fig. 4; Table S1).

10.1128/mBio.03238-19.2 TABLE S1 Growth curve raw data. Download Table S1, XLSX file, 0.1 MB. Copyright © 2020 Ito et al. 2020 Ito et al. This content is distributed under the terms of the Creative Commons Attribution 4.0 International license.

### Competitive colonization assays in gnotobiotic mice.

**FIG 5:**
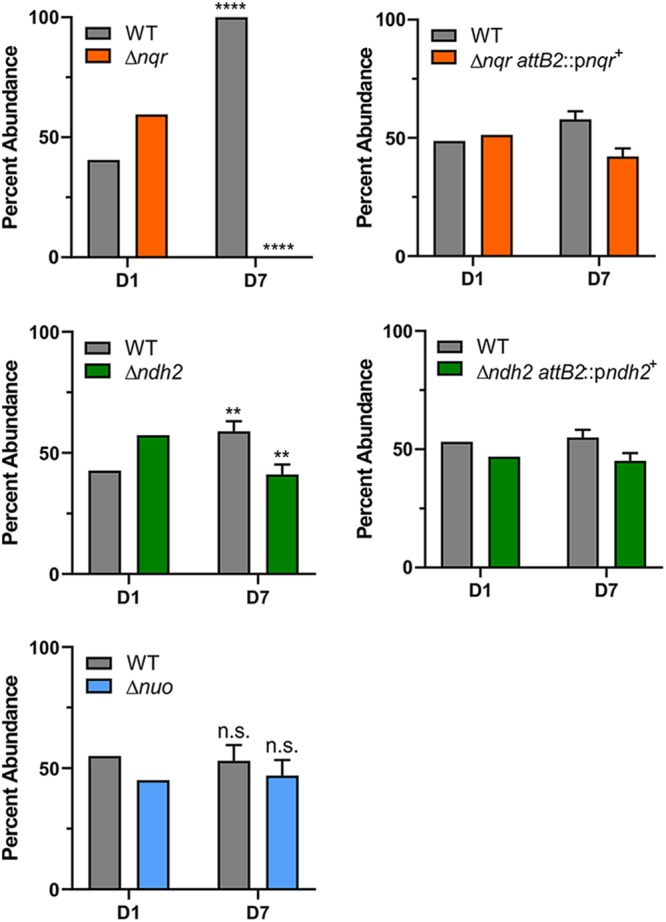
Mouse competitive colonization experiments with the B. fragilis WT and respiration mutants show fitness defects of the Δ*nqr* and Δ*ndh2* mutants. Data are representative results for the B. fragilis WT and respiration mutant Δ*nqr*, Δ*ndh2*, and Δ*nuo* strains or complemented Δ*nqr attB*::p*nqr^+^* and Δ*ndh2 attB*::p*ndh2^+^* strains in mouse gut colonization assays. The percentage of WT and mutant strains in the inoculum (day 1 [D1]) and feces (day [D7]) of the samples is shown as the mean and standard error of the mean (when applicable). The differences in abundance of the Δ*nqr* and the Δ*ndh2* mutants from days 1 to 7 were statistically significant (****, *P = *1E−40; **, *P = *0.0002), but they were not for the Δ*nuo* mutant (*P > *0.05 [n.s., not significant]). All experiments were performed with 3 male and 3 female germfree Swiss Webster mice. The phenotype was replicated in both the males and the females, and the breakdown by sex is shown in Fig. S1 in the supplemental material.

As the *nqr*, *ndh2*, and *nuo* single deletion mutants did not demonstrate any significant *in vitro* growth defect compared to the growth of the WT, we tested their importance to bacterial fitness *in vivo*. For each mutant, competitive colonization assays (competing WT with the mutant) were performed using gnotobiotic mice (Fig. 5; Table S2). After a week of competitive colonization, we found that the Δ*nqr* mutant was outcompeted and was present as less than 1% of the total bacteria in the feces, as no Δ*nqr* mutants were detected in the more than 100 colonies analyzed for each mouse. The ability of this mutant to competitively colonize was reestablished to a large extent, although not completely, when the *nqr* operon was restored to the Δ*nqr* mutant. In contrast, the Δ*nuo* mutant showed no significant difference in colonization from that of the WT in the competitive colonization assay, while the Δ*ndh2* mutant showed a modest colonization defect, with the mutant colonization being reduced from 57.3% in the starting inoculum to 41.1% ± 4.1% (standard deviation) in the feces at 1 week (*P = *0.0002). This colonization defect was also reversed when *ndh2* was added back to this mutant (Fig. 5). These data support the findings of the activity assays showing that NQR is the most active NADH:quinone oxidoreductase and the most important enzyme complex of the three studied for bacterial colonization in this model system.

10.1128/mBio.03238-19.3 TABLE S2 Mouse competitive colonization raw data. Download Table S2, XLSX file, 0.01 MB. Copyright © 2020 Ito et al. 2020 Ito et al. This content is distributed under the terms of the Creative Commons Attribution 4.0 International license.

10.1128/mBio.03238-19.1 FIG S1 Results of mammalian gut competitive colonization assays of the B. fragilis WT and respiration mutants by sex of the mice. All experiments were performed with 3 male and 3 female germfree Swiss Webster mice. The same inoculum was used for both males and females. The percentages of WT and mutant strains in the inoculum (day 1 [D1]) and feces (day 7 [D7]) of the samples are graphed with the mean and standard error of the mean (when applicable). Phenotypes were similar in both sexes. Download FIG S1, TIF file, 0.9 MB. Copyright © 2020 Ito et al. 2020 Ito et al. This content is distributed under the terms of the Creative Commons Attribution 4.0 International license.

## DISCUSSION

**FIG 6:**
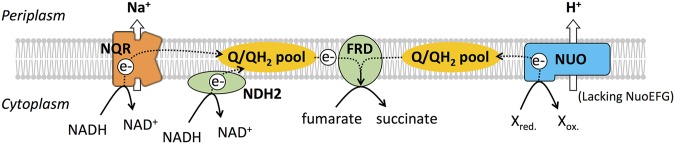
Current model of anaerobic respiration in B. fragilis. Substrate reactions and electron transfers are shown as solid and broken arrows, respectively. NQR and NDH2 oxidize NADH to NAD^+^ and donate electrons to the menaquinone pool (Q/QH_2_). Fumarate reductase (FRD) accepts electrons from reduced menaquinone and transfers them to the terminal electron acceptor, fumarate, which is reduced to succinate. B. fragilis NUO, lacking the NADH binding domain (NuoEFG subunits), likely oxidizes an unknown electron donor (X_red._) to transfer electrons via the menaquinone pool to FRD. In this scheme, energy is conserved when ion gradients are generated by NQR and NUO.

In this study, we defined the enzymes involved in the crucial first step of respiration in B. fragilis, the transfer of electrons from NADH to quinone (Fig. 6). While all three typical classes of NADH dehydrogenases are present in B. fragilis, we found that only two have this activity. Under the growth conditions used in this study, NQR was the most active and the most important NADH:quinone oxidoreductase. Deletion of *nqr* resulted in a mutant that was severely attenuated for competitive colonization of the mouse intestine. A second enzyme, NDH2, could also catalyze this step and similarly had a competitive colonization defect, albeit not as severe as that of the Δ*nqr* mutant. In addition, B. fragilis has a homolog of complex I (NUO) for which we could not demonstrate NADH:quinone oxidoreductase activity, nor could we demonstrate a fitness defect of the Δ*nuo* mutant in the mouse colonization model.

The lack of NADH:quinone oxidoreductase activity of NUO may be attributable to poor expression of the *nuo* operon under the conditions of our assay. Our analysis of published transcriptome sequencing data (62) revealed that the *nuo* operon is in fact poorly expressed during anaerobic growth in rich medium. However, the lack of NADH oxidation activity is likely also explained by NUO’s unusual subunit composition. In comparison to the *nuo* operons of most bacteria, the *nuoE*, *nuoF*, and *nuoG* genes are missing in B. fragilis (Fig. 1). In a typical NUO complex, NuoE, NuoF, and NuoG make up a hydrophilic domain that includes the conserved NADH binding site. A NUO lacking this soluble domain should not be able to use NADH as a substrate. We analyzed 144 B. fragilis genomes and found that all have similar *nuo* operons lacking these three genes. The conservation of this operon in the species suggests that it has a different yet likely still important function in respiration. NUO complexes lacking these subunits have been described. The enzyme complex from Prevotella copri has the same 11-subunit organization as that from B. fragilis (52), while the enzyme from Campylobacter jejuni does not have NuoE and NuoF but retains NuoG, giving it a total of 12 subunits (52, 63). The more typical form of NUO, which uses NADH as a substrate, is exemplified by the complex from E. coli, with 14 subunits, including NuoE, NuoF, and NuoG (51). We predict that the B. fragilis NUO receives electrons from a distinct donor and transfers them to menaquinone while generating an H^+^ motive force (Fig. 6).

NQR is found only in prokaryotes and is present in many marine and pathogenic bacteria. In most organisms where NQR has been studied, the enzyme operates as a primary Na^+^ pump (64, 65). Our findings suggest that *Bacteroides* generates an Na^+^ gradient directly through respiration by NQR, rather than depending completely on Na^+^/H^+^ antiporters to generate an Na^+^ gradient. The primacy of NQR as the most active NADH:quinone oxidoreductase in B. fragilis and as the most important one for competitive colonization suggests that the Na^+^ gradient likely has a key role in the production and distribution of energy in these bacteria. In fact, more than 10 genes annotated as encoding Na^+^-dependent transporters are present in the B. fragilis 638R genome.

B. fragilis is also capable of aerobic respiration, employing the terminal oxidase cytochrome *bd*, which is expected to conserve energy in the form of an H^+^ electrochemical membrane gradient (49, 57). We predict that nanaerobic respiration confers a fitness advantage in the oxygenated environment near the colonic mucus layer. Thus, to fully understand the respiratory pathways in B. fragilis and their importance in energy conservation under different environmental conditions in the gut, it will be important to also better define the properties and enzymes of this nanaerobic respiratory chain.

## MATERIALS AND METHODS

### Bacterial strains and growth conditions.

For all studies, we used a strain of B. fragilis 638R (TM4000) and its *thy*-negative mutant (ADB77). The strains were grown anaerobically at 37°C in brain heart infusion supplemented (BHIS) medium containing 0.5% (wt/vol) yeast extract, 5 μg/ml hemin, and, when necessary, 50 μg/ml thymine, as well as antibiotics (5 μg/ml erythromycin, 200 μg/ml gentamicin, or 40 ng/ml anhydrotetracycline [aTC]). Escherichia coli strain S17 λ *pir* and derivatives were grown in LB broth or plates, with 100 μg/ml ampicillin or carbenicillin being added where appropriate. All strains and plasmids used are listed in Table S3 in the supplemental material.

10.1128/mBio.03238-19.4 TABLE S3 Strains and plasmids used or created in this study. Download Table S3, DOCX file, 0.02 MB. Copyright © 2020 Ito et al. 2020 Ito et al. This content is distributed under the terms of the Creative Commons Attribution 4.0 International license.

### Construction of deletion mutant strains.

**(i) *nqr* (BF638R_2136-2141) and *nuo* (BF638R_0841-0850) operon deletion.** Upstream and downstream flanking regions of the *nqr* and *nuo* operons were amplified by PCR using the primers listed in Table S4 containing BamHI and NcoI sites for the upstream fragment and NcoI and HindIII sites for the downstream portion. These PCR products were digested and cloned by three-way ligation into BamHI-HindIII-digested pTY102 (66). The resulting plasmids were introduced into B. fragilis ADB77 by conjugation. Cointegrates were selected with erythromycin and gentamicin, and the cointegrate was passaged in nonselective medium and then plated on plates with 80 to 100 μg/ml trimethoprim and 50 μg/ml thymine to select against cointegrates (66). The resulting colonies were tested by PCR to differentiate mutant and WT resolvants. For animal experiments, the *thy* mutation in the background strain was restored to the WT by allelic exchange with the *thy*-positive suicide vector pYT102 (66).

10.1128/mBio.03238-19.5 TABLE S4 Primers used in this study. Download Table S4, DOCX file, 0.01 MB. Copyright © 2020 Ito et al. 2020 Ito et al. This content is distributed under the terms of the Creative Commons Attribution 4.0 International license.

**(ii) Creation of pLGB36, an inducible counterselection vector for allelic deletions and replacements in B. fragilis 638R.** Plasmid pLGB36 was made specifically to make allelic deletions and replacements in B. fragilis 638R and other B. fragilis strains that have the same type VI secretion system (T6SS) locus as strain 638R (67). This vector was created using the backbone of plasmid pLGB13 (68), which was designed for allelic replacements in *Bacteroides* and *Parabacteroides* species using the type VI secretion effector Bfe1 of strain 638R. As pLEC13 uses the 638R effector, strains with this T6SS region are immune to this toxin. In pLGB36, we used an effector from the T6SS region of B. fragilis strain S36_L11 (M136_1999), which we designated *bfe-3*, and replaced *bfe-1* and its engineered signal sequence of pLGB13 with *bfe-3* with the same signal sequence to target the effector to the periplasm, where it is toxic upon induction with aTC. We have not found any resistance to this toxin, nor have we encountered the growth of any colonies that are not double-cross-out resolvants, demonstrating the utility of this vector for allelic deletions and replacements in strain 638R and others encoding the Bfe1 effector/immunity pair.

**(iii) *ndh2* (BF638R_1612) deletion.** The *ndh2* deletion was created using pLGB36. Upstream and downstream flanking regions were cloned into the BamHI site of pLGB36 using the NEBuilder assembly tool (New England Biolabs) with the primers listed in Table S4 and transformed into E. coli S17 λ *pir*. The construct was verified by whole-plasmid sequencing and transferred by conjugation into TM4000. Cointegrates were selected on BHIS medium plates with gentamicin and erythromycin. A cointegrate was grown in basal medium (69) for 5 h and then plated with 40 ng/μl aTC. Resolvants were screened by PCR and mutants were selected.

### Complementation of mutant strains.

**(i) *nqr* operon complementation.** The complete *nqr* operon with its native promoter (6,563 bp) was amplified by PCR using primers with a NotI restriction site at the 5′ end and a BamHI restriction site at the 3′ end (Table S4). The digested PCR product was cloned into NotI- and BamHI-digested pNBU2-*bla*-*ermGb*, which integrates into the chromosome and is tranformed into S17 λ *pir*. The construct was sequenced to confirm that no mutations were introduced. The plasmid was transferred by conjugation into the *nqr* deletion strain. Transconjugants with integration of the plasmid at the *attB2* site of tRNA-Ser were selected by PCR using the primers listed in Table S4.

**(ii) *ndh2* complementation.** The *ndh2* gene with its promoter was cloned into pNBU2-*bla*-*ermGb* using the NEBuilder assembly tool with the primers listed in Table S4 and transformed into S17 λ *pir*. The construct was verified by whole-plasmid sequencing. Transconjugants with integration of the plasmid at the *attB2* site of tRNA-Ser were selected by PCR, as described above.

### Cell membrane preparation.

All bacterial cultures were harvested in mid-logarithmic growth phase and washed with KPi buffer, containing 40 mM KH_2_PO_4_, pH 7, and 5 mM dithiothreitol. The cells were broken using a French press (24,000 lb/in^2^, 2 cycles) under anaerobic conditions in KPi buffer containing DNase and phenylmethylsulfonyl fluoride (PMSF) protease inhibitor at 4°C. Broken cells were removed by centrifugation (3,800 × *g*), and the remaining supernatant was then centrifuged at 185,500 × *g* overnight to separate the inner membranes. The inner membrane preparations were washed and resuspended in KPi buffer under anaerobic conditions and stored at −80°C.

### NADH:quinone oxidoreductase activity.

The NADH:quinone oxidoreductase activity was followed spectrophotometrically at room temperature under an argon atmosphere in a buffer containing 50 mM Tris-HCl, pH 7, 1 mM EDTA, 100 mM NaCl, 5% (vol/vol) glycerol, 0.05% (wt/vol) *n*-dodecyl β-maltoside, 100 μM NADH, and 50 μM menadione (vitamin K_3_). As it has been reported that menaquinone is the quinone produced by B. fragilis (70), menadione was used as the electron acceptor from NADH for all activity studies.

The oxidation of K_2_-NADH or nicotinamide hypoxanthine dinucleotide, reduced-form Na^+^ salt (deamino-NADH), was measured at 343 nm (ε = 6.22 mM^−1^ cm^−1^), where menadione and menadiol are isosbestic (71). The reduction of menadione was measured at 262 nm (ε = 14.0 mM^−1^ cm^−1^), and the results were adjusted by subtracting the calculated contribution of the accumulating NAD^+^ from the total absorbance at 262 nm. The absorbance of NAD^+^ at 262 nm (ε = 17.8 mM^−1^ cm^−1^) was calculated from the absorbance change at 343 nm. Membrane preparations from the WT or mutant strains were used at a protein concentration of 60 μg/ml. The reaction was initiated by adding membranes to the buffer containing 100 μM NADH and 50 μM vitamin K_3_ under an argon atmosphere. All activity measurements were performed at least three times.

### Growth analysis.

Cultures of the wild-type and mutant strains described in this paper were grown in BHIS broth overnight, diluted 1 to 10 in fresh broth, and grown to mid-exponential phase. A suitable volume (usually 10 μl) was then used to inoculate 1 ml of prereduced BHIS broth in wells of a 24-well tissue culture plate. All incubations were at 37°C under anaerobic conditions. After 1 min of shaking, OD_600_ readings were recorded every 10 min for 12 h using a BioTek Power Wave plate reader (all OD_600_ data and doubling time calculations are provided in Table S1).

Doubling times were calculated for each strain on a segment of the logarithmic growth phase using the exponential growth equation [*y* = *y*_0_·exp(*k*·*x*)], with *y* being OD_600_, *y*_0_ being OD_600_ at time zero, and *k* being rate constant expressed in inversed minutes, with a least-squares fit, as implemented in Prism software (64-bit version 8.2.0 for Windows; GraphPad Software, Inc., San Diego, CA), and are presented as the average from at least two experiments (see the details in Table S1). The OD_600_ data were graphed using Prism software after the data were transformed by averaging the values for 5 neighbors and using zero-order smoothing.

### Competitive colonization assays in gnotobiotic mice.

Mouse studies were approved by the Institutional Animal Care and Use Committee (IACUC) of Brigham and Women’s Hospital and complied with all relevant ethical regulations for animal testing and research. Gnotobiotic mice were obtained from and maintained in the Harvard Digestive Diseases Center gnotobiotic core facility at Brigham and Women’s Hospital and housed in sterile OptiMICE cages (Animal Care Systems, Centennial, CO). All experiments were performed using Swiss Webster germfree mice that were 5 to 8 weeks old. For each experiment, both male and female mice (groups of three male and three female mice) were used. To differentiate the strains for quantification, the WT B. fragilis TM4000 strain was made tetracycline resistant by introduction of pNBU2-*bla*-*tetQ* into the *attB2* tRNA-Ser site, and the mutant strains were made erythromycin resistant by integration of pNBU2-*bla*-*ermGb*. For experiments with complemented mutant strains, the pNBU2-*bla*-*ermGb* constructs containing the cloned genes for complementation were similarly integrated into the *attB2* tRNA-Ser site. Strains were grown to an OD_600_ of approximately 0.6 and mixed at a 1:1 ratio, with the final percentage of each strain in the inoculum being determined by plating, as reported in Fig. 5 and Table S2. Mice were gavaged with 200 μl of the bacterial mixtures, and 7 days later, fresh fecal samples were collected, diluted in sterile phosphate-buffered saline, and plated on BHIS medium plates. After colony growth, the bacteria were replica plated onto two plates containing either tetracycline or erythromycin to determine the exact percentage of each strain in the feces (Fig. 5; Table S2). A one-sample *t* test was performed using the arcsine-transformed values of the proportions of the WT and mutant strains present in the samples on days 1 and 7. The Δ*nqr* mutant competition data set had no standard error since there were no Δ*nqr* mutant colonies detected, so the value 1E−13 was added to one sample so that the values of the *t* test could be computed.

### Data availability.

Plasmid pLGB36 has been deposited in Addgene under accession no. 135621 for distribution to the scientific community.

## References

[1] Blaser MJ, Cardon ZG, Cho MK, Dangl JL, Donohue TJ, Green JL, Knight R, Maxon ME, Northen TR, Pollard KS, Brodie EL 2016 Toward a predictive understanding of Earth’s microbiomes to address 21st century challenges. mBio 7:e00714-16. doi:10.1128/mBio.00714-16. 27178263 PMC4895116

[2] McGlynn SE, Chadwick GL, Kempes CP, Orphan VJ 2015 Single cell activity reveals direct electron transfer in methanotrophic consortia. Nature 526:531–535. doi:10.1038/nature15512. 26375009

[3] Stacy A, Fleming D, Lamont RJ, Rumbaugh KP, Whiteley M, Stacy A, Fleming D, Lamont RJ, Rumbaugh KP, Whiteley M 2016 A commensal bacterium promotes virulence of an opportunistic pathogen via cross-respiration. mBio 7:e00782-16. 27353758 10.1128/mBio.00782-16 PMC4916382

[4] Hammer ND, Reniere ML, Cassat JE, Zhang Y, Hirsch AO, Hood MI, Skaar EP 2013 Two heme-dependent terminal oxidases power *Staphylococcus aureus* organ-specific colonization of the vertebrate host. mBio 4:e00241-13. doi:10.1128/mBio.00241-13. 23900169 PMC3735196

[5] Lan L, Cheng A, Dunman PM, Missiakas D, He C 2010 Golden pigment production and virulence gene expression are affected by metabolisms in *Staphylococcus aureus*. J Bacteriol 192:3068–3077. doi:10.1128/JB.00928-09. 20400547 PMC2901709

[6] Rivera-Chávez F, Zhang LF, Faber F, Lopez CA, Byndloss MX, Olsan EE, Xu G, Velazquez EM, Lebrilla CB, Winter SE, Bäumler AJ 2016 Depletion of butyrate-producing *Clostridia* from the gut microbiota drives an aerobic luminal expansion of *Salmonella*. Cell Host Microbe 19:443–454. doi:10.1016/j.chom.2016.03.004. 27078066 PMC4832419

[7] Lopez CA, Kingsbury DD, Velazquez EM, Bäumler AJ 2014 Collateral damage: microbiota-derived metabolites and immune function in the antibiotic era. Cell Host Microbe 16:156–163. doi:10.1016/j.chom.2014.07.009. 25121745 PMC4151313

[8] Louis P, Hold GL, Flint HJ 2014 The gut microbiota, bacterial metabolites and colorectal cancer. Nat Rev Microbiol 12:661–672. doi:10.1038/nrmicro3344. 25198138

[9] Varel VH, Bryant MP 1974 Nutritional features of *Bacteroides fragilis* subsp. *fragilis*. Appl Microbiol 28:251–257. 4853401 10.1128/am.28.2.251-257.1974 PMC186696

[10] Hooper LV, Stappenbeck TS, Hong CV, Gordon JI 2003 Angiogenins: a new class of microbicidal proteins involved in innate immunity. Nat Immunol 4:269–273. doi:10.1038/ni888. 12548285

[11] Hooper LV, Littman DR, Macpherson AJ 2012 Interactions between the microbiota and the immune system. Science 336:1268–1273. doi:10.1126/science.1223490. 22674334 PMC4420145

[12] Kau AL, Ahern PP, Griffin NW, Goodman AL, Gordon JI 2011 Human nutrition, the gut microbiome and the immune system. Nature 474:327–336. doi:10.1038/nature10213. 21677749 PMC3298082

[13] Mayer EA, Knight R, Mazmanian SK, Cryan JF, Tillisch K 2014 Gut microbes and the brain: paradigm shift in neuroscience. J Neurosci 34:15490–15496. doi:10.1523/JNEUROSCI.3299-14.2014. 25392516 PMC4228144

[14] Nicholson JK, Holmes E, Kinross J, Burcelin R, Gibson G, Jia W, Pettersson S 2012 Host-gut microbiota metabolic interactions. Science 336:1262–1267. doi:10.1126/science.1223813. 22674330

[15] Stappenbeck TS, Hooper LV, Gordon JI 2002 Developmental regulation of intestinal angiogenesis by indigenous microbes via Paneth cells. Proc Natl Acad Sci U S A 99:15451–15455. doi:10.1073/pnas.202604299. 12432102 PMC137737

[16] Tang WH, Hazen SL 2014 The contributory role of gut microbiota in cardiovascular disease. J Clin Invest 124:4204–4211. doi:10.1172/JCI72331. 25271725 PMC4215189

[17] Faith JJ, Guruge JL, Charbonneau M, Subramanian S, Seedorf H, Goodman AL, Clemente JC, Knight R, Heath AC, Leibel RL, Rosenbaum M, Gordon JI 2013 The long-term stability of the human gut microbiota. Science 341:1237439. doi:10.1126/science.1237439. 23828941 PMC3791589

[18] Koropatkin NM, Cameron EA, Martens EC 2012 How glycan metabolism shapes the human gut microbiota. Nat Rev Microbiol 10:323–335. doi:10.1038/nrmicro2746. 22491358 PMC4005082

[19] Poole RK, Cook GM 2000 Redundancy of aerobic respiratory chains in bacteria? Routes, reasons and regulation. Adv Microb Physiol 43:165–224. doi:10.1016/s0065-2911(00)43005-5. 10907557

[20] Kerscher S, Dröse S, Zickermann V, Brandt U 2008 The three families of respiratory NADH dehydrogenases. Results Probl Cell Differ 45:185–222. doi:10.1007/400_2007_028. 17514372

[21] Trumpower BL, Gennis RB 1994 Energy transduction by cytochrome complexes in mitochondrial and bacterial respiration: the enzymology of coupling electron transfer reactions to transmembrane proton translocation. Annu Rev Biochem 63:675–716. doi:10.1146/annurev.bi.63.070194.003331. 7979252

[22] Kröger A, Geisler V, Lemma E, Theis F, Lenger R 1992 Bacterial fumarate respiration. Arch Microbiol 158:311–314. doi:10.1007/BF00245358.

[23] Meehan BM, Malamy MH 2012 Fumarate reductase is a major contributor to the generation of reactive oxygen species in the anaerobe *Bacteroides fragilis*. Microbiology 158:539–546. doi:10.1099/mic.0.054403-0. 22075026 PMC3352283

[24] Frigaard NU, Dahl C 2009 Sulfur metabolism in phototrophic sulfur bacteria. Adv Microb Physiol 54:103–200. doi:10.1016/S0065-2911(08)00002-7. 18929068

[25] Stewart V 1988 Nitrate respiration in relation to facultative metabolism in enterobacteria. Microbiol Rev 52:190–232. 3045516 10.1128/mr.52.2.190-232.1988 PMC373136

[26] Barquera B, Hellwig P, Zhou W, Morgan JE, Häse CC, Gosink KK, Nilges M, Bruesehoff PJ, Roth A, Lancaster CRD, Gennis RB 2002 Purification and characterization of the recombinant Na^+^-translocating NADH:quinone oxidoreductase from *Vibrio cholerae*. Biochemistry 41:3781–3789. doi:10.1021/bi011873o. 11888296

[27] Crofts AR, Hong S, Ugulava N, Barquera B, Gennis R, Guergova-Kuras M, Berry EA 1999 Pathways for proton release during ubihydroquinone oxidation by the bc(1) complex. Proc Natl Acad Sci U S A 96:10021–10026. doi:10.1073/pnas.96.18.10021. 10468555 PMC17835

[28] Friedrich T, Stolpe S, Schneider D, Barquera B, Hellwig P 2005 Ion translocation by the *Escherichia coli* NADH:ubiquinone oxidoreductase (complex I). Biochem Soc Trans 33:836–839. doi:10.1042/BST0330836. 16042610

[29] Wikström M 2004 Cytochrome c oxidase: 25 years of the elusive proton pump. Biochim Biophys Acta 1655:241–247. doi:10.1016/j.bbabio.2003.07.013. 15100038

[30] Sazanov LA 2015 A giant molecular proton pump: Structure and mechanism of respiratory complex I. Nat Rev Mol Cell Biol 16:375–388. doi:10.1038/nrm3997. 25991374

[31] Hunte C, Palsdottir H, Trumpower BL 2003 Protonmotive pathways and mechanisms in the cytochrome bc_1_ complex. FEBS Lett 545:39–46. doi:10.1016/s0014-5793(03)00391-0. 12788490

[32] Konings WN 2006 Microbial transport: adaptations to natural environments. Antonie Van Leeuwenhoek 90:325–342. doi:10.1007/s10482-006-9089-3. 17043914

[33] Jung H 2001 Towards the molecular mechanism of Na^+^/solute symport in prokaryotes. Biochim Biophys Acta 1505:131–143. doi:10.1016/S0005-2728(00)00283-8. 11248195

[34] Maloney PC, Kashket ER, Wilson TH 1974 A protonmotive force drives ATP synthesis in bacteria. Proc Natl Acad Sci U S A 71:3896–3900. doi:10.1073/pnas.71.10.3896. 4279406 PMC434292

[35] Dimroth P 1994 Bacterial sodium ion-coupled energetics. Antonie Van Leeuwenhoek 65:381–395. doi:10.1007/bf00872221. 7832594

[36] Schneider D, Pohl T, Walter J, Dorner K, Kohlstadt M, Berger A, Spehr V, Friedrich T 2008 Assembly of the *Escherichia coli* NADH:ubiquinone oxidoreductase (complex I). Biochim Biophys Acta 1777:735–739. doi:10.1016/j.bbabio.2008.03.003. 18394423

[37] Efremov RG, Sazanov LA 2011 Structure of the membrane domain of respiratory complex I. Nature 476:414–420. doi:10.1038/nature10330. 21822288

[38] Baradaran R, Berrisford JM, Minhas GS, Sazanov LA 2013 Crystal structure of the entire respiratory complex I. Nature 494:443–448. doi:10.1038/nature11871. 23417064 PMC3672946

[39] Reyes-Prieto A, Barquera B, Juárez O 2014 Origin and evolution of the sodium pumping NADH:quinone oxidoreductase. PLoS One 9:e96696. doi:10.1371/journal.pone.0096696. 24809444 PMC4014512

[40] Barquera B 2014 The sodium pumping NADH:quinone oxidoreductase (Na^+^-NQR), a unique redox-driven ion pump. J Bioenerg Biomembr 46:289–298. doi:10.1007/s10863-014-9565-9. 25052842

[41] Belevich NP, Bertsova YV, Verkhovskaya ML, Baykov AA, Bogachev AV 2016 Identification of the coupling step in Na^+^-translocating NADH:quinone oxidoreductase from real-time kinetics of electron transfer. Biochim Biophys Acta 1857:141–149. doi:10.1016/j.bbabio.2015.12.001. 26655930

[42] Juárez O, Morgan JE, Nilges MJ, Barquera B 2010 Energy transducing redox steps of the Na^+^-pumping NADH:quinone oxidoreductase from *Vibrio cholerae*. Proc Natl Acad Sci U S A 107:12505–12510. doi:10.1073/pnas.1002866107. 20616050 PMC2906589

[43] Heikal A, Nakatani Y, Dunn E, Weimar MR, Day CL, Baker EN, Lott JS, Sazanov LA, Cook GM 2014 Structure of the bacterial type II NADH dehydrogenase: a monotopic membrane protein with an essential role in energy generation. Mol Microbiol 91:950–964. doi:10.1111/mmi.12507. 24444429

[44] Caspari D, Macy JM 1983 The role of carbon dioxide in glucose metabolism of *Bacteroides fragilis*. Arch Microbiol 135:16–24. doi:10.1007/bf00419476. 6414431

[45] Macy JM, Probst I 1979 The biology of gastrointestinal *Bacteroides*. Annu Rev Microbiol 33:561–594. doi:10.1146/annurev.mi.33.100179.003021. 386933

[46] Macy JM, Ljungdahl LG, Gottschalk G 1978 Pathway of succinate and propionate formation in *Bacteroides fragilis*. J Bacteriol 134:84–91. 148460 10.1128/jb.134.1.84-91.1978 PMC222221

[47] Macy JM, Probst I, Gottschalk G 1975 Evidence for cytochrome involvement in fumarate reduction and adenosine 5′ triphosphate synthesis by *Bacteroides fragilis* grown in the presence of hemin. J Bacteriol 123:436–442. 1150622 10.1128/jb.123.2.436-442.1975 PMC235746

[48] Baughn AD, Malamy MH 2003 The essential role of fumarate reductase in haem-dependent growth stimulation of *Bacteroides fragilis*. Microbiology 149:1551–1558. doi:10.1099/mic.0.26247-0. 12777495

[49] Baughn AD, Malamy MH 2004 The strict anaerobe *Bacteroides fragilis* grows in and benefits from nanomolar concentrations of oxygen. Nature 427:441–444. doi:10.1038/nature02285. 14749831

[50] Van Hellemond JJ, Tielens A 1994 Expression and functional properties of fumarate reductase. Biochem J 304:321–331. doi:10.1042/bj3040321. 7998964 PMC1137495

[51] Moparthi VK, Hägerhäll C 2011 The evolution of respiratory chain complex I from a smaller last common ancestor consisting of 11 protein subunits. J Mol Evol 72:484–497. doi:10.1007/s00239-011-9447-2. 21597881 PMC3144371

[52] Franke T, Deppenmeier U 2018 Physiology and central carbon metabolism of the gut bacterium *Prevotella copri*. Mol Microbiol 109:528–540. doi:10.1111/mmi.14058. 29995973

[53] Deusch S, Scheicher L, Seifert J, Steuber J 2019 Occurrence and function of the Na^+^ translocating NADH:quinone oxidoreductase in *Prevotella* spp. Microorganisms 7:e117. doi:10.3390/microorganisms7050117. 31035603 PMC6560451

[54] Duran-Pinedo AE, Nishikawa K, Duncan MJ 2007 The RprY response regulator of *Porphyromonas gingivalis*. Mol Microbiol 64:1061–1074. doi:10.1111/j.1365-2958.2007.05717.x. 17501928

[55] Li Y, Krishnan K, Duncan MJ 2018 Post-translational regulation of a *Porphyromonas gingivalis* regulator. J Oral Microbiol 10:1487743. doi:10.1080/20002297.2018.1487743. 29988788 PMC6032018

[56] Albenberg L, Esipova TV, Judge CP, Bittinger K, Chen J, Laughlin A, Grunberg S, Baldassano RN, Lewis JD, Li H, Thom SR, Bushman FD, Vinogradov SA, Wu GD 2014 Correlation between intraluminal oxygen gradient and radial partitioning of intestinal microbiota in humans and mice. Gastroenterology 147:1055–1063. doi:10.1053/j.gastro.2014.07.020. 25046162 PMC4252572

[57] Puustinen A, Finel M, Haltia T, Gennis RB, Wikström M 1991 Properties of the two terminal oxidases of *Escherichia coli*. Biochemistry 30:3936–3942. doi:10.1021/bi00230a019. 1850294

[58] Borisov VB, Gennis RB, Hemp J, Verkhovsky MI 2011 The cytochrome bd respiratory oxygen reductases. Biochim Biophys Acta 1807:1398–1413. doi:10.1016/j.bbabio.2011.06.016. 21756872 PMC3171616

[59] Matsushita K, Ohnishi T, Kaback HR 1987 NADH-ubiquinone oxidoreductases of the *Escherichia coli* aerobic respiratory chain. Biochemistry 26:7732–7737. doi:10.1021/bi00398a029. 3122832

[60] Zambrano MM, Kolter R 1993 *Escherichia coli* mutants lacking NADH dehydrogenase I have a competitive disadvantage in stationary phase. J Bacteriol 175:5642–5647. doi:10.1128/jb.175.17.5642-5647.1993. 8366049 PMC206622

[61] Zhou WD, Bertsova YV, Feng BT, Tsatsos P, Verkhovskaya ML, Gennis RB, Bogachev AV, Barquera B 1999 Sequencing and preliminary characterization of the Na^+^-translocating NADH:ubiquinone oxidoreductase from *Vibrio harveyi*. Biochemistry 38:16246–16252. doi:10.1021/bi991664s. 10587447

[62] Chatzidaki-Livanis M, Geva-Zatorsky N, Comstock LE 2016 *Bacteroides fragilis* type VI secretion systems use novel effector and immunity proteins to antagonize human gut *Bacteroidales* species. Proc Natl Acad Sci U S A 113:3627–3632. doi:10.1073/pnas.1522510113. 26951680 PMC4822612

[63] Häse CC, Barquera B 2001 Role of sodium bioenergetics in *Vibrio cholerae*. Biochim Biophys Acta 1505:169–178. doi:10.1016/S0005-2728(00)00286-3. 11248198

[64] Häse CC, Fedorova ND, Galperin MY, Dibrov PA 2001 Sodium ion cycle in bacterial pathogens: evidence from cross-genome comparisons. Microbiol Mol Biol Rev 65:353–370. doi:10.1128/MMBR.65.3.353-370.2001. 11528000 PMC99031

[65] Weerakoon DR, Olson JW 2008 The *Campylobacter jejuni* NADH:ubiquinone oxidoreductase (complex I) utilizes flavodoxin rather than NADH. J Bacteriol 190:915–925. doi:10.1128/JB.01647-07. 18065531 PMC2223568

[66] Baughn AD, Malamy MH 2002 A mitochondrial-like aconitase in the bacterium *Bacteroides fragilis*: implications for the evolution of the mitochondrial Krebs cycle. Proc Natl Acad Sci U S A 99:4662–4667. doi:10.1073/pnas.052710199. 11880608 PMC123704

[67] Coyne MJ, Roelofs KG, Comstock LE 2016 Type VI secretion systems of human gut Bacteroidales segregate into three genetic architectures, two of which are contained on mobile genetic elements. BMC Genomics 17:58. doi:10.1186/s12864-016-2377-z. 26768901 PMC4714493

[68] Garcia-Bayona L, Comstock LE 2019 Streamlined genetic manipulation of diverse *Bacteroides* and *Parabacteroides* isolates from the human gut microbiota. mBio 10:e01762-19. doi:10.1128/mBio.01762-19. 31409684 PMC6692515

[69] Coyne MJ, Béchon N, Matano LM, McEneany VL, Chatzidaki-Livanis M, Comstock LE 2019 A family of anti-Bacteroidales peptide toxins wide-spread in the human gut microbiota. Nat Commun 10:3460. doi:10.1038/s41467-019-11494-1. 31371723 PMC6671954

[70] Ramotar K, Conly JM, Chubb H, Louie TJ 1984 Production of menaquinones by intestinal anaerobes. J Infect Dis 150:213–218. doi:10.1093/infdis/150.2.213. 6470528

[71] Sheppard CA, Trimmer EE, Matthews RG 1999 Purification and properties of NADH-dependent 5,10-methylenetetrahydrofolate reductase (MetF) from *Escherichia coli*. J Bacteriol 181:718–725. 9922232 10.1128/jb.181.3.718-725.1999 PMC93435

