# Supplementary figures and images for: Genetic and Biochemical Analysis of Anaerobic Respiration in Bacteroides fragilis and Its Importance In Vivo
Source: mBio. 2020 Feb 4;11(1):e03238-19. doi: 10.1128/mBio.03238-19 (PMC7002350; doi:10.1128/mBio.03238-19)

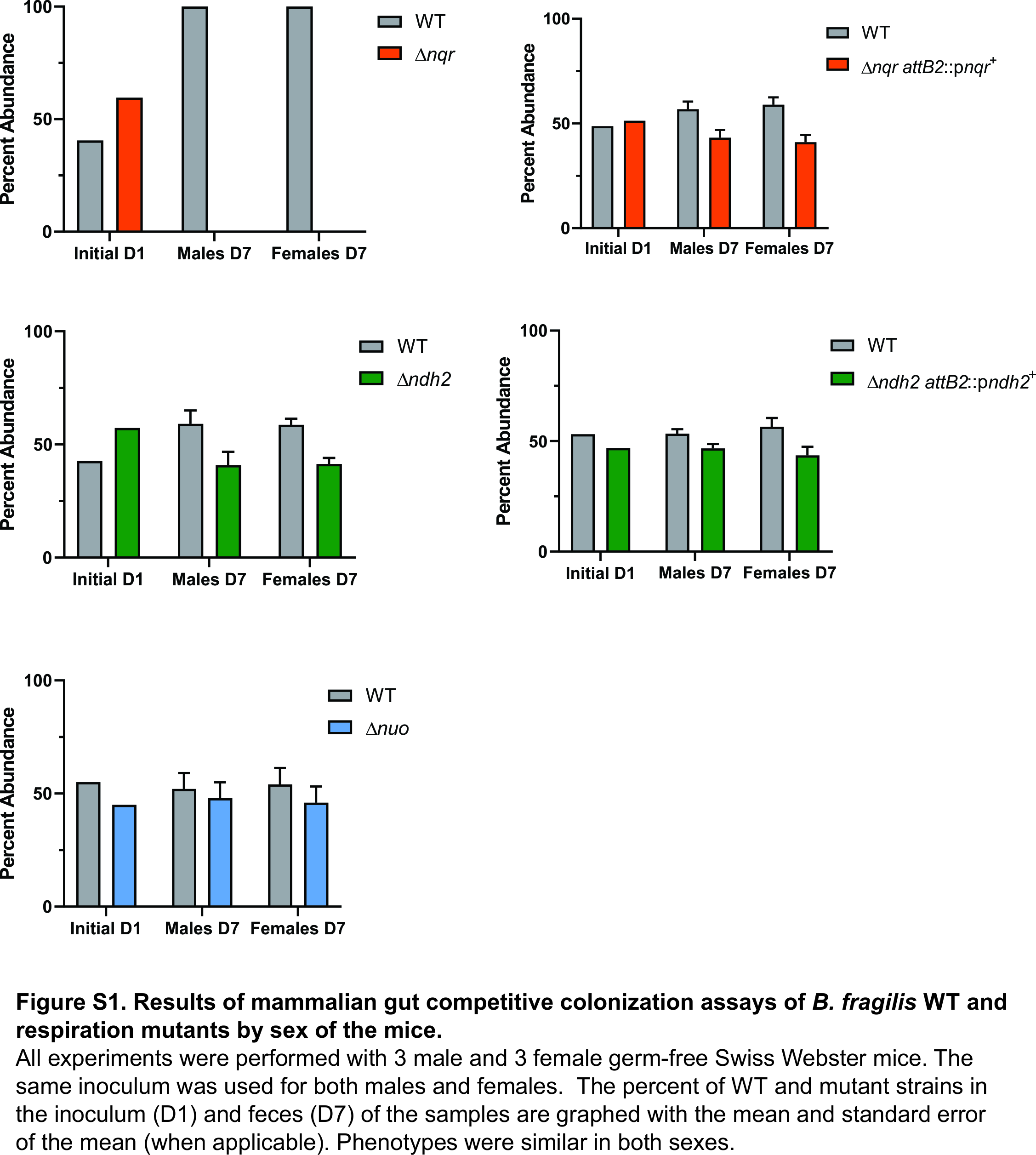

Supplement: FIG S1 [file mBio.03238-19-sf001.tif]
