# Supplementary material for: Genetic and Biochemical Analysis of Anaerobic Respiration in Bacteroides fragilis and Its Importance In Vivo
Source: mBio. 2020 Feb 4;11(1):e03238-19. doi: 10.1128/mBio.03238-19 (PMC7002350; doi:10.1128/mBio.03238-19)
Supplement: TABLE S3 [file mBio.03238-19-st003.docx]

**Table S3. Strains and plasmids used or created in this study**

| **Name** | **Description** | **Reference/Source** |
| --- | --- | --- |
| **STRAINS** |  |  |
| *Escherichia coli* |  |  |
| S17 λ pir  DH5α  HB101 | (F− ) RP4-2-Tc::Mu *aphA*::Tn*7recAλpir* lysogen  λ non-lysogen  F^-^ Δ(*mcrC*-*mrr*)*recA13rpsL20* | Simon *et al*. 1983.  NEB  Sambrook *et al*.  1989 |
| *Bacteroides fragilis* |  |  |
| BF638R (TM4000) | Clinical Isolate, Rif^R^ | clinical isolate,Pasteur Institute, Madeleine Sebald as 638rfm |
| ADB77 | TM4000 Δ*thyA* | Baughn and Malamy, 2002 |
| ADB77 Δ*nqr* * | ADB77 Δ*nqrA,B,C,D,E,F* | This work |
| ADB77 Δ*nuo* * | ADB77 Δ*nuoA,B,C,H,I,J,K,L,M,N* | This work |
| TM4000 Δ*ndh2* | TM4000 Δ*ndh2* | This work |
| ADB77 Δ*nqr* Δ*nuo* * | ADB77 Δ*nqr*, Δ*nuo* | This work |
| ADB77 Δ*nqr* Δ*ndh2 ** | ADB77 Δ*nqr*, Δ*ndh2* | This work |
| ADB77 Δ*nuo* Δ*ndh2* | ADB77 *nuo*, Δ*ndh2* | This work |
| ADB77 Δ*nqr* *attB2*::p*nqr^+^* | ADB77 Δnqr::pRAG473 | This work |
| TM4000 Δ*ndh2* *attB2*::p*ndh2^+^* | TM4000 Δndh2:: p*ndh2^+^* | This work |
| ADB77 Δ*nqr* Δ*ndh2attB2*::p*ndh2^+*^* | ADB77 Δ*nqr* Δndh2 :: p*ndh2^+^* | This work |
|  |  |  |
| **PLASMIDS** |  |  |
| pLGB36 | Constructed for deletions in *B. fragilis* 638R. Derivative of pLGB13 with M136_2283 (*bfe3*) replacing *bfe1* | This work |
|  |  |  |
| pNBU2-*bla*-*ermGb* |  | Koropatkin  *et al*. 2008 |
| pNBU2-*bla*-*tetQ* |  |  |
| RK231 | RP4 derivative, Kan^R^Tet ^R^, Tra^+^ | Guiney *et al*. 1984 |
| pYT102 | p15A *ori*, Cm^R^, RP4 *oriT*, B.fragilis suicide vector containing *B.fragilis thyA*^+^ Tet^R^ | Tang and Malamy, 2000 |
| pMKK01 | Δ*nqrA,B,C,D,E,F* deletion plasmid; pYT102 | This work |
| pMKK02 | Δ*nuoA,B,C,H,I,J,K,L,M,N* deletion plasmid; pYT102 | This work |
| p*ndh2* | Flanking regions of the *ndh2* gene in pLGB36. *bla* (Ec)*, erm* (bf) | This work |
| p*ndh2*^+^ | *ndh2* and promoter gene cloned into pNBU2-*bla-ermGb.* *bla* (Ec)*, erm* (Bf) | This work |
| pRAG473 | *nqr* and promoter gene cloned into pNBU2-*bla-ermGb.* *bla* (Ec)*, erm* (Bf) | This work |

* These deletions were originally created in ADB77 then converted to thy^+^ by marker rescue with pYT102.

-Simon R, Priefer U, Puhler, A 1983. A broad host range mobilization system for *in vivo* genetic engineering: transposon mutagenesis in Gram negative bacteria. Biotechnology **1:**784–791.

-Sambrook,J., E. Fritsch and T. Maniatis. 1989. Molecular cloning: a laboratory manual, 2nd ed. Cold Spring Harbor Laboraory Press, Cold Spring Harbor, NY

-Baughn AD, Malamy MH. 2002. A mitochondrial-like aconitase in the bacterium *Bacteroides fragilis*: Implications for the evolution of the mitochondrial Krebs cycle. Proc Natl Acad Sci 99:4662–4667.

-Koropatkin NM, Martens EC, Gordon JI, Smith TJ. 2008. Starch catabolism by a prominent human gut symbiont is directed by the recognition of amylose helices. Structure 16:1105-1115.

-Guiney DG, Hasegawa P, Davis CE.1984. Plasmids transfer from *Escherichia coli* to *Bacteroides fragilis*: differential expression of antibiotic resistance phenotypes. PNAS U S A. 81:7203-7206.

-Tang, YP, Malamy MH. 2000. Isolation of *Bacteroides fragilis* mutants with in vivo growth defects by using Tn4400', a modified Tn4400 transposition system and a new screening method. Infect. Immun. 68:415-419.
