# Supplementary material for: Genetic and Biochemical Analysis of Anaerobic Respiration in Bacteroides fragilis and Its Importance In Vivo
Source: mBio. 2020 Feb 4;11(1):e03238-19. doi: 10.1128/mBio.03238-19 (PMC7002350; doi:10.1128/mBio.03238-19)
Supplement: TABLE S4 [file mBio.03238-19-st004.docx]

**Table S4. Primers used in this study**

| **Primers** | **Sequence** | |
| --- | --- | --- |
| **Deletion of *nqr*** | |  |
| left_*nqr*-fwd | | TTGGGATCCACATGTTCCGCCTGGTAGTG |
| left_*nqr*-rev | | GAGTCCATGGAATCCCGGTTCTTTCACCGT |
| right_*nqr*_fwd | | CTGCCCATGGAGCCGGTGTGAAGTATACGG |
| right_*nqr*_rev | | CCGAAGCTTGTCGGGCAACTGTCCGATAA |
| **Deletion of *nuo*** | |  |
| left_*nuo*_fwd | | GATGGATCCAGAAGGAAGAGGGTGTGGAG |
| left_*nuo*_rev | | GTATCCATGGTTGCATCTATCAGGGAATTGACAAG |
| right_*nuo*_fwd | | CACCCCATGGACAAAGGCAATTGCGGTCAG |
| right_*nuo*_rev | | TCCAAGCTTCCGGAATATCCTGGAACGCT |
| **Deletion of *ndh2*** | |  |
| left_*ndh2*_fwd | | Ggcatagtatcagatgagtggattcctggcacgcgaac |
| left_*ndh2*_rev | | Gttgttgcgtgttgaaactcatactgaaaacttctatttaacc |
| right_*ndh2*_fwd | | Gagtttcaacacgcaacaacccaaaggc |
| rigth_*ndh2*_rev | | Cgaattcctgcagcccggggcggattatattgatgcttatggactg |
| **Complementation of *nqr*** | |  |
| *nqr*_comp_fwd | | TGCGCGGCCGCGCTTCCTTCCGTCATCTTCTT |
| *nqr*_comp_rev | | TCCGATATCCCTTGAACACCTGCTCTATCTGA |
| **Complementation of *ndh2*** | |  |
| *ndh2*_comp_fwd | | cggccgctctagaactagtggaaaccgttcttttagctg |
| *ndh2*_ comp_rev | | cgaattcctgcagcccgggggtgaactttagcctttgg |
| ***att*B2 integration site screen** | |  |
| Bf-*att*B2-tRNA-Ser-fwd | | tgattcgtacctttgcatcg |
| Bf-*att*B2-tRNA-Ser-rev | | ggcagattgttgctttggtt |
| **pLGB36 construct** | |  |
| pLGB13 no *bfe1*_fwd | | gtggatcccccgggctgc |
| pLGB13 no *bfe1*_rev | | gtgtcttttcttttatatgtctttatttcgtctctatcactg |
| ss-bfe3 _fwd | | acatataaaagaaaagacacatgaagaaaattttatctttgc |
| ss-bfe3_rev | | ctgcagcccgggggatccactcatctgatactatgccaatg |

Restriction sites are underlined
